# Elexacaftor/tezacaftor/ivacaftor’s effects on cystic fibrosis infections are maintained, but not increased, after 3.5 years of treatment

**DOI:** 10.1172/JCI184171

**Published:** 2024-09-05

**Authors:** Sarah J. Morgan, Ellis Coulter, Hannah L. Betts, George M. Solomon, John P. Clancy, Steven M. Rowe, David P. Nichols, Pradeep K. Singh, the PROMISE-Micro Study Group

**Affiliations:** 1 University of Washington, Seattle, Washington, USA.; 2 University of Alabama, Birmingham, Alabama, USA.; 3 Cystic Fibrosis Foundation, Bethesda, Maryland, USA.; 4 The PROMISE-Micro Study Group is detailed in Supplemental Acknowledgments.

**Keywords:** Microbiology, Bacterial infections

**To the Editor:** Elexacaftor/tezacaftor/ivacaftor (ETI) is the most effective modulator drug currently available for most people with cystic fibrosis (PwCF) (1). Data after 0.5–1 year of use show that ETI rapidly reduces sputum CF-pathogen density, although most PwCF remain infected by the bacteria present prior to treatment (1, 2). The durability of ETI-associated infection responses are uncertain, as bacterial genetic or phenotypic adaptations could reverse the improvements in pathogen density. Indeed, our previous work on the first-available modulator (ivacaftor) found that pathogen density rebounded after approximately 1–2 years in a small cohort of adults with relatively advanced lung disease (3).

Here, we studied sputum collected by the prospective 27-center PROMISE-Micro study to test the hypothesis that ETI produces durable effects on the prevalence and sputum density of CF pathogens. We analyzed microbiological outcomes through 3.5 years of ETI treatment from 177 participants (see demographics in Supplemental Table 1; supplemental material available online with this article; https://doi.org/10.1172/JCI184171DS1) providing sputum before and after ETI. Data from the first 6 months, consent, methods, and cohort clinical responses were detailed previously (1). Study limitations are described in Nichols et al. (1) and include limitations inherent to studying sputum, reduced sputum production after treatment, and droplet-digital PCR’s (ddPCR’s) inability to distinguish live from dead bacteria. Notably, participants reported decreased use of other medicines that could improve infection outcomes after ETI initiation, including inhaled antibiotics, hypertonic saline, and recombinant human DNase (Supplemental Figure 1A).

We began by measuring the prevalence of pathogen-positive sputum cultures to gauge cohort-level infection burden. *Staphylococcus aureus* (*Sa*) prevalence decreased at 2.5 years (16.2% relative reduction, *P =* 0.0125) but not at other time points (Figure 1A). *Pseudomonas aeruginosa* (*Pa*), and *Stenotrophomonas maltophilia* (*Sm*) prevalence decreased after 1 month (*Pa* relative reduction 24.8%, *P =* 0.0009; *Sm* relative reduction 61.3%, *P =* 0.0009), but not further through 3.5 years (Figure 1A).

Eradicating infections is highly desirable, but difficult to prove using sputum. We measured the proportion of baseline culture-positive participants that had 3 or more consecutive culture- and ddPCR-negative samples, including the last provided sample, as a proxy for infection clearance. Only 1.3% (1 of 79) baseline *Sa*-positive and 10% (5 of 50) baseline *Pa*-positive participants with sufficient sputum for both analyses became repeatedly negative (Figure 1B). In contrast, 50% (11 of 22) of baseline *Sm*-positive participants became repeatedly negative. More participants were negative by culture alone (i.e., ignoring ddPCR results) (Figure 1B).

We previously found that a similar proportion of participants became culture negative after 6 months (1); thus, 3.5 years of ETI did not substantially increase the proportion of participants becoming repeatedly negative. Notably, the probability of a participant becoming repeatedly *Pa* negative showed an inverse relationship with baseline *Pa* density (Supplemental Figure 1B). In addition, average baseline *Sm* density was approximately 50-fold lower than average baseline *Sa* and *Pa* density, and more participants became *Sm* negative (Figure 1B). Both observations suggest that ETI-improved host defenses may be more effective when pathogen burden is low.

We examined changes in pathogen culture density in baseline-positive participants. Cohort-wide average *Sa* density decreased by 2.06 and *Pa* by 2.1 log_10_CFU/g at 1 month (*P* < 0.001) and showed additional declines at 1.5 years (*Sa* by –0.7 and *Pa* by –1.1 log_10_CFU/g vs. 1 month [*P* < 0.009]) (Figure 1, C and E). These reductions were generally maintained through 3.5 years. *Sm* decreased by 0.7 log_10_CFU/g at 1 month (*P* < 0.001) without additional change (Figure 1G). *Achromobacter* and *Burkholderia* spp. showed similar patterns, although fewer participants cultured these organisms (Supplemental Figure 2).

These cohort-wide data include samples that were transiently or repeatedly culture negative, and negative samples could mask bacterial density increases in persistently infected participants. To test this, we eliminated culture-negative samples and still found no rebound in *Sa*, *Pa*, or *Sm* density (Figure 1, D, F, and H). Importantly, average post-ETI pathogen densities in persistently positive samples were approximately 10- to 1000-fold higher than overall cohort-wide averages (that included culture-negative samples). These more modest pathogen reductions better represent ETI responses in persistently infected participants.

Finally, we gauged the frequency of “new” infections by examining participants who were culture negative in the 2 years before ETI by registry report and culture negative at baseline. We found that 36.4% (8 of 22) of previously *Sa*-negative, 11.9% (8 of 67) previously *Pa*-negative, and 10.3% (12 of 116) previously *Sm*-negative participants became culture positive at least once during 3.5 years of ETI treatment (Supplemental Figure 3A). A minority of these participants were repeatedly culture positive (Supplemental Figure 3B), and culture densities were low (Supplemental Figure 3, C–E).

This interim analysis had 4 main findings. First, unlike after ivacaftor (3), a rebound in pathogen density was not apparent through 3.5 years of ETI treatment. One explanation is that bacterial adaptation is strongly influenced by the residual bacterial population size (i.e., smaller populations→fewer mutations→slower adaptation). Consistent with this explanation, the persistently infected ETI-treated participants studied here had approximately 10-fold lower sputum *Pa* densities after ETI than the ivacaftor-treated cohort (3), perhaps due to a milder disease in the ETI-treated cohort and higher drug efficacy. Importantly, smaller bacterial populations can still adapt, but do so more slowly. Thus, a rebound in pathogen density could occur in the future.

Second, the analyses removing culture-negative samples showed that average *Pa* and *Sm* density decreased modestly in persistently infected participants. However, pathogen density decreases (i.e., CFU/g) in sputum may not reflect changes in total lung pathogen burden, and it is unknown whether either parameter predicts future lung disease.

Third, most of the decrease in pathogen density occurred after 1 month, with little further improvement. This could be explained if the pathogen populations infecting PwCF consisted of subpopulations that were rapidly ETI responsive and subpopulations relatively unresponsive (e.g., because of their location or physiological state), if organisms adapted to new conditions, or if reduced use of other medications counteract some of ETI’s beneficial effects.

Fourth, people who are apparently uninfected with pathogens before ETI can develop at least transient infections while taking ETI. These infections could be clinically insignificant. Alternatively, they could lead to chronic infections and lung damage, particularly if host defenses degrade due to aging, progression of CF or other lung diseases, or environmental insults. Continued follow-up and mechanistic research could address these questions.

## Supplementary Material

Supplemental data

Supporting data values

## Figures and Tables

**Figure 1:**
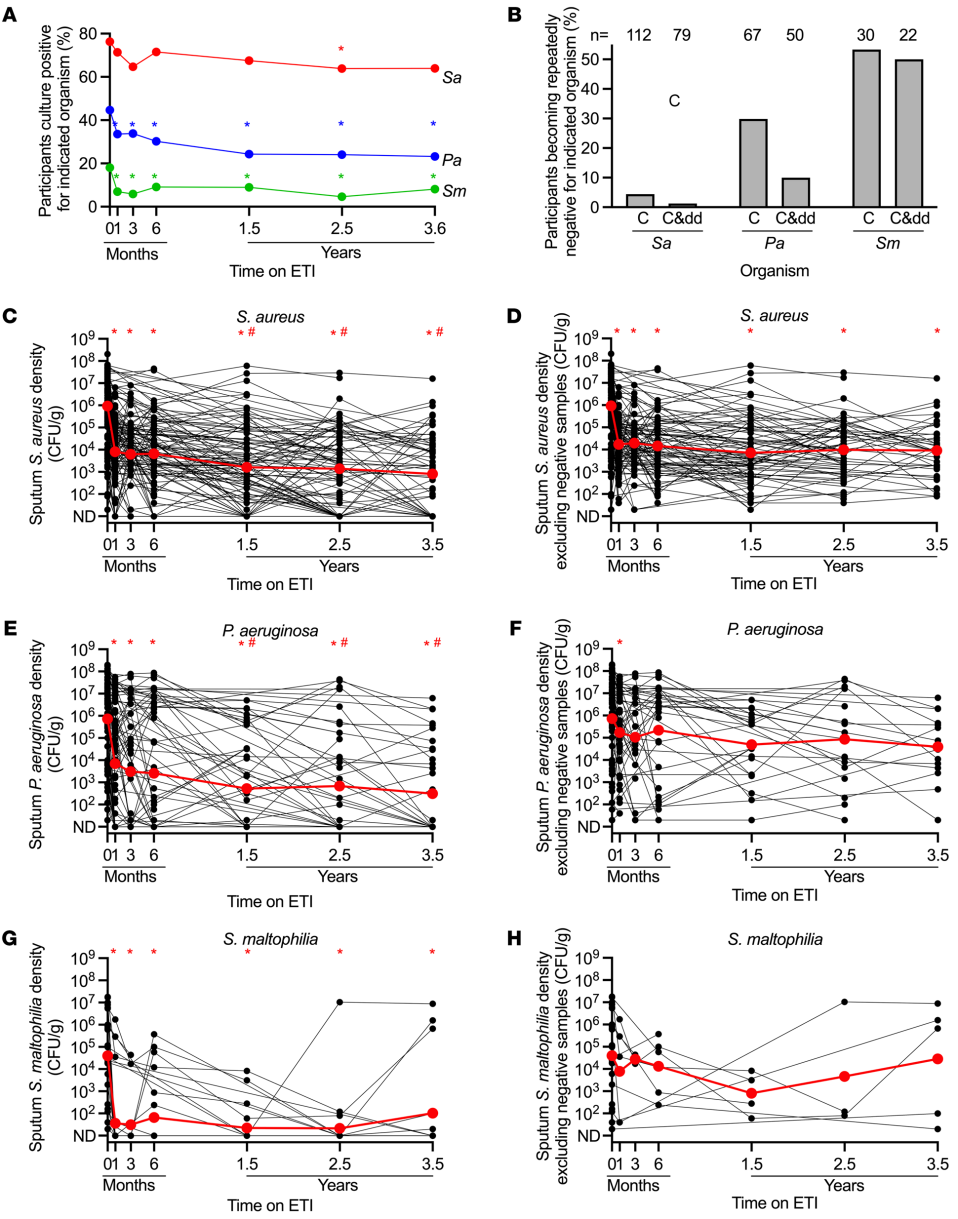
Sputum microbiology after ETI. (**A**) Proportion of participants culture positive for indicated pathogens (includes participants missing data at visit). *P < 0.05 by McNemar’s exact test. (**B**) Proportion of baseline-positive participants becoming repeatedly negative for indicated pathogen by culture (C) or culture and ddPCR (C&dd); *n* = number of participants analyzed. (**C**–**H**) Culture density in participants who were baseline culture positive for indicated pathogens including (**C**, **E**, and **G**) and not including (**D**, **F**, and **H**) culture-negative samples. Similar differences were apparent between all-data vs. data with culture-negative samples eliminated in by-participant averages (Supplemental Figure 1, C–H). Individual participants are indicated in black, averages in red. The limit of detection = 20 CFU/g. *P < 0.05 vs. baseline; ^#^*P* < 0.05 vs. 1 month by mixed-effects analysis (**C**–**H**).

## Version 1 09/05/2024

In-Press Preview

## Version 2 10/15/2024

Electronic publication
