# Supplementary material for: Elexacaftor/tezacaftor/ivacaftor’s effects on cystic fibrosis infections are maintained, but not increased, after 3.5 years of treatment
Source: J Clin Invest. 2024 Sep 5;134(20):e184171. doi: 10.1172/JCI184171 (PMC11473140; doi:10.1172/JCI184171)
Supplement: Supplemental data [file jci-134-184171-s155.pdf]

Supplemental Methods:

Sex as a biological variable: Both male and female participants were involved in this study. Females enrolled at slightly higher levels and therefore comprised 52.5% of the participants.

Statistics: The proportion of participants with a given pathogen at each timepoint was analyzed by McNemar's exact test. Significant changes in pathogen abundance were determined using mixed-effects analysis comparing pre-ETI to post-ETI timepoints and one month on ETI to later post-ETI timepoints. Summary statistics including mean, standard deviation, and proportion were used to describe the baseline characteristics of the cohort. All statistics were performed in Graphpad PRISM.

Study approval: Institutional review board approval was granted at each individual participating site. The study was registered at ClinicalTrials.gov (NCT04038047).

Data availability: Data are available in the "Supporting data values" XLS file.

Supplemental Acknowledgements
This study was supported by the US Cystic Fibrosis Foundation and National Institute of Health grant R01HL148274 (P.S.). The content of this manuscript is the responsibility of the authors alone and does not necessarily reflect the views or policies of the study sponsor. Special thanks to the individuals with CF and their families who participated in this study, and whose dedication to research made the trial possible. We also thank the CF Data Safety Monitoring Board (DSMB) with Data Monitoring Committee (DMC) led by Dr. Lynne Quittell for study oversight, and the Center for CF Microbiology (L. Hoffman, Director) for processing some sputum samples.

Table S1. Demographics and Baseline Characteristics by visits attended.

		All enrolled participants (N = 236)*	Baseline and at least one post-ETI (n=177)	Baseline and at least 3 post-ETI (n= 127)
Age (years), mean (SD)		24.8 (10.9)	25.5 (10.8)	25.67 (10.6)
Age (years) distribution, n (%)	12 to < 18	85 (36.0%)	54 (30.5%)	40 (31.5%)
	18 to < 30	79 (33.5%)	63 (35.6%)	42 (33.1%)
	30 or older	72 (30.5%)	60 (33.9%)	45 (35.4%)
Sex, n (%)	Male	112 (47.5%)	80 (45.2%)	55 (43.3%)
	Female	124 (52.5%)	97 (54.8%)	72 (56.7%)
Race, n (%)	White	220 (93.2%)	164 (92.7%)	118 (92.9%)
	Black or African American	4 (1.7%)	4 (2.3%)	2 (1.6%)
	Asian	0 (0%)	0 (0%)	0 (0%)
	American Indian or Alaska Native	0 (0%)	0 (0%)	0 (0%)
	Native Hawaiian or Other Pacific Islander	1 (0.4%)	1 (0.5%)	1 (0.8%)
	More than One Race	9 (3.8%)	7 (4.0%)	5 (3.9%)
	Unknown/Missing	2 (0.8%)	1 (0.6%)	1 (0.8%)
Hispanic or Latino, n (%)	Yes	17 (7.2%)	13 (7.3%)	9 (7.1%)
	No	219 (92.8%)	164 (92.7%)	118 (92.9%)
ppFEV, mean (SD)		80.4 (22.4)	78.41 (22.5)	78.6 (23.0%)
ppFEV distribution, n (%)	< 65	63 (26.7%)	53 (29.9%)	38 (29.9%)
	65 to 90	83 (35.2%)	62 (35.0%)	44 (34.6%)
	> 90	90 (38.1%)	62 (35.0%)	45 (35.4%)
Sweat Chloride (mmol/L), mean (SD)		87.5 (17.9)	88.0 (18.1)	87.8 (18.7%)
Height (cm) [18+ y.o.], mean (SD)		167.2 (9.4)	166.9 (9.01)	166.9 (8.6%)
Weight (kg) [18+ y.o.], mean (SD)		65.0 (13.7)	60.8 (14.1)	62.5 (14.6%)
BMI (kg/m2) [18+ y.o.], mean (SD)		23.2 (4.1)	22.2 (4.1)	22.8 (4.4%)
Genotype Group, n (%)	F508del Homozygous	113 (47.9%)	87 (49.2%)	65 (51.2%)
	F508del Heterozygous (MF)	97 (41.1%)	70 (39.5%)	47 (37.0%)
	F508del Heterozygous (G551D)	14 (5.9%)	10 (5.6%)	7 (5.5%)
	F508del Heterozygous (other)	12 (5.1%)	10 (5.6%)	8 (6.3%)
Prior Modulator Use, n (%)	None	119 (50.4%)	85 (48.0%)	58 (45.7%)
	Orkambi or Symdeko	104 (44.1%)	81 (45.8%)	62 (48.8%)
	Kalydeco	13 (5.5%)	11 (6.2%)	7 (5.5%)
Pathogens detected pre-ETI, n (%)	None detected	7 (3.4%)	6 (3.4%)	4 (3.1%)
	Achromobacter spp.	12 (5.8%)	12 (6.8%)	8 (6.3%)
	Burkholderia spp.	8 (3.9%)	8 (4.5%)	6 (4.7%)
	Pseudomonas aeruginosa	90 (43.7%)	79 (44.6%)	57 (44.9%)
	Staphylococcus aureus	154 (74.8%)	135 (76.3%)	95 (74.8%)
	Stenotrophomonas maltophilia	39 (18.9%)	32 (18.1%)	26 (20.5%)

* Data on all enrolled participants previously published in (1)

Figure S1

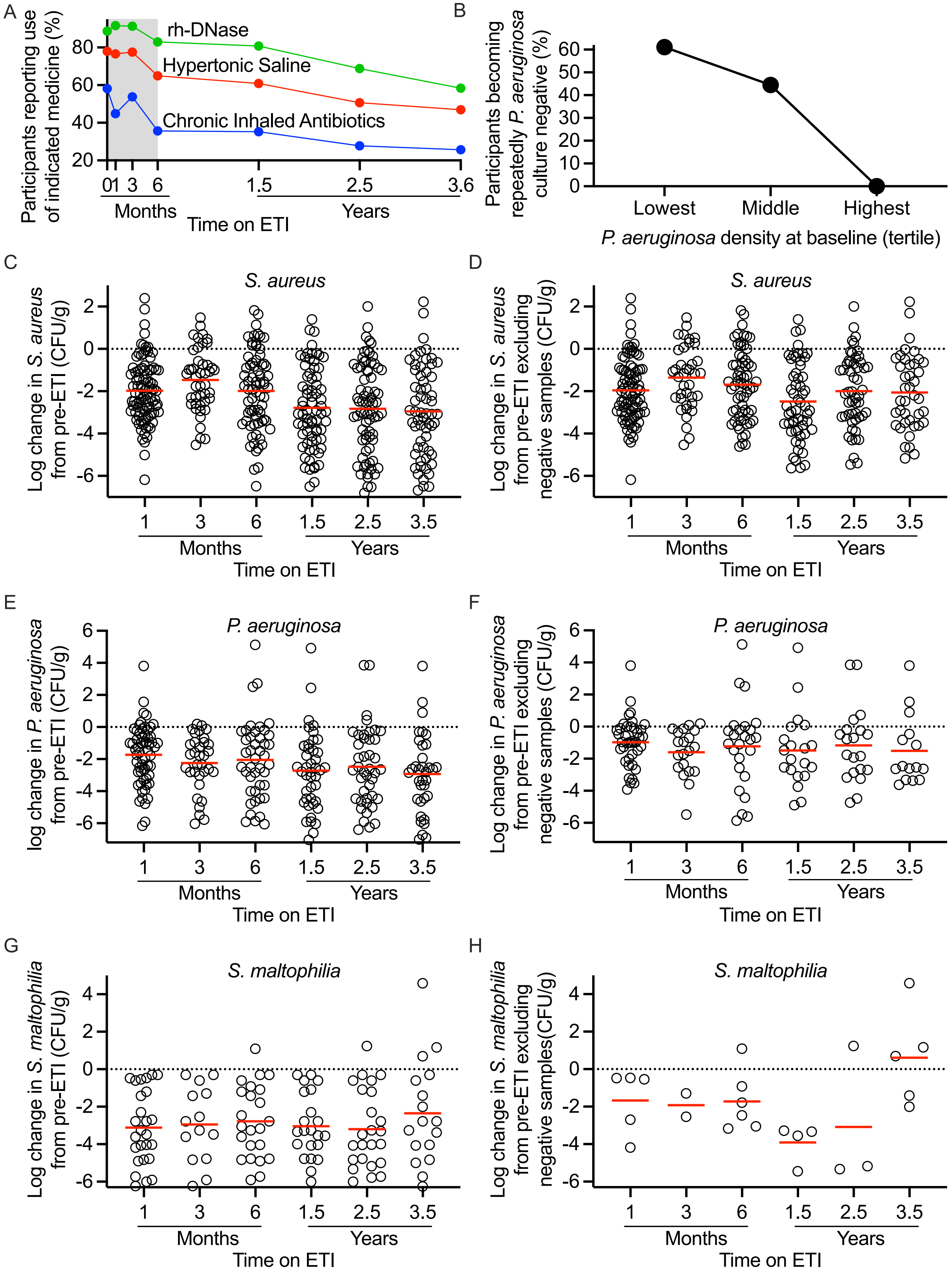
A
Participants reporting use of indicated medicine (%)
rh-DNase
Hypertonic Saline
Chronic Inhaled Antibiotics
Months
Years
Time on ETI
B
Participants becoming repeatedly P. aeruginosa culture negative (%)
Lowest
Middle
Highest
P. aeruginosa density at baseline (tertile)
C
S. aureus
Log change in S. aureus from pre-ETI (CFU/g)
D
Log change in S. aureus from pre-ETI excluding negative samples (CFU/g)
E
P. aeruginosa
log change in P. aeruginosa from pre-ETI (CFU/g)
F
Log change in P. aeruginosa from pre-ETI excluding negative samples (CFU/g)
G
S. maltophilia
Log change in S. maltophilia from pre-ETI (CFU/g)
H
Log change in S. maltophilia from pre-ETI excluding negative samples(CFU/g)

Figure S1. Pathogen response to ETI

A. Proportion of participants reporting use of indicated medication at each visit. At enrollment, participants were asked to maintain prescribed treatments through the first 6 months of the study (grey shaded area).

B. Proportion of participants becoming repeatedly culture-negative is highest among participants with the lowest baseline Pa density. Baseline Pa density was calculated by tertial; lowest tertial had baseline Pa sputum density less than $1x10^5$ CFU/g and highest density tertial had baseline Pa sputum greater than $1.1x10^7$ CFU/g. Plot shows percentage of participants in each tertial that became repeatedly-culture negative. Repeatedly culture negative was defined as participants for whom at least the last three sputum samples provided (post-ETI) were culture negative. To be included in this analysis participants had to be Pa positive pre-ETI and provided at least three sputum samples post-ETI. Importantly, Pa densities of samples in the lowest baseline Pa density tertile group generally far exceeded the limit of detection. Thirteen of 18 of the samples in this group had baseline Pa densities 10-fold higher than the limit of detection and 10/18 samples had baseline Pa densities 100-fold higher than the limit of detection. Thus, it is unlikely conversion to repeatedly culture-negative status in the lowest tertile group was due to false-negative results that can sometime occur near detection limits.

C-G. By-participant average pathogen responses to ETI. Change in CFU/g in participants who were baseline culture-positive for indicated pathogens, including (B, D, F) and not including (C, E, G) culture-negative samples. To calculate log changes, log-transformed pre-ETI CFU/g values were subtracted from the post-ETI CFU/g values for each participant. Individual participants are indicated in black, averages in red. The limit of detection was 20 CFU/g.

Figure S2.

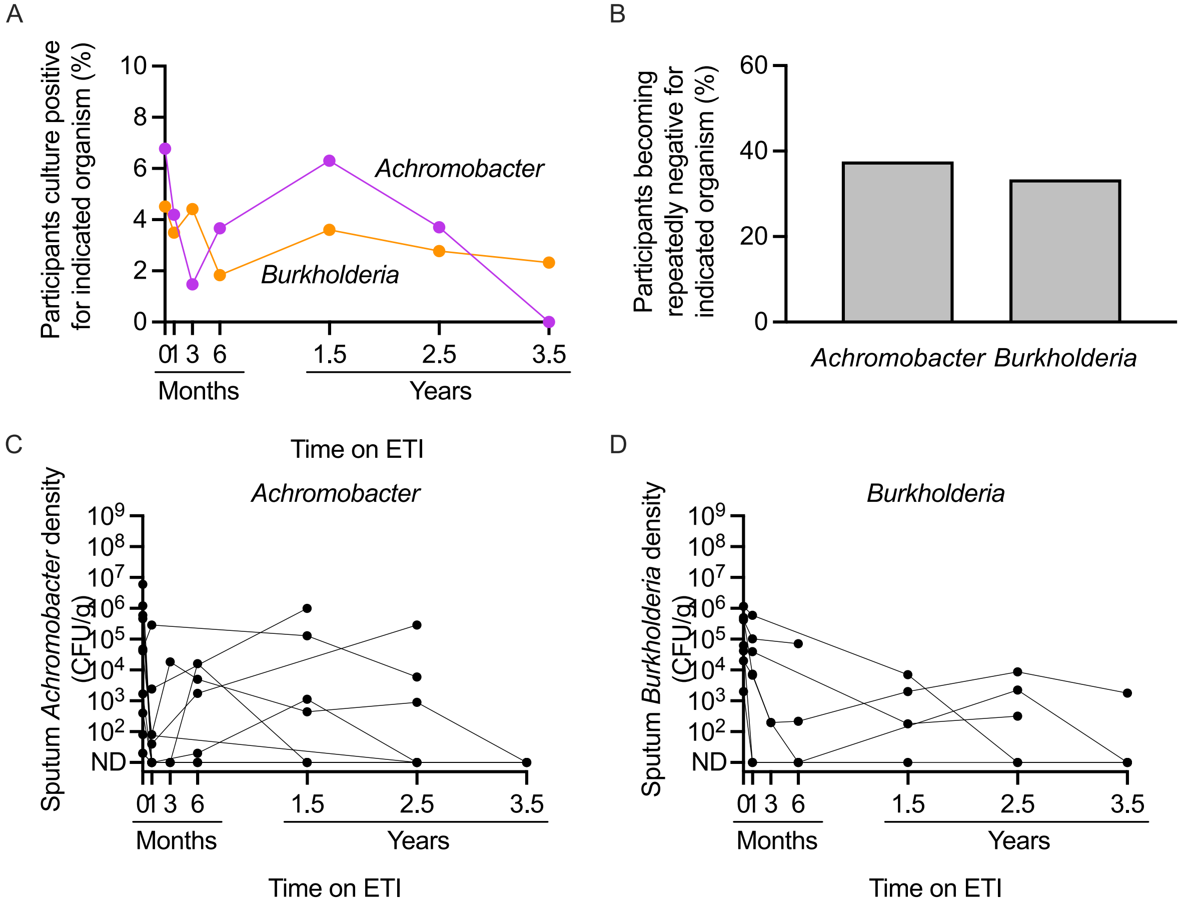

Figure S2. Changes in *Achromobacter* and *Burkholderia sp.* cultures after ETI. A. Proportion of participants sputum culture-positive for indicated organism (includes participants missing data at one or more visit). **B.** Proportion of baseline culture-positive participants becoming repeatedly negative for indicated pathogens by culture (ddPCR not used for these organisms). **C-D**. Culture density of indicated pathogens in participants who were culture-positive for the indicated pathogen at baseline. Individual participants are indicated in black and linked by a line. The limit of detection was 20 CFU/g.

Figure S3.

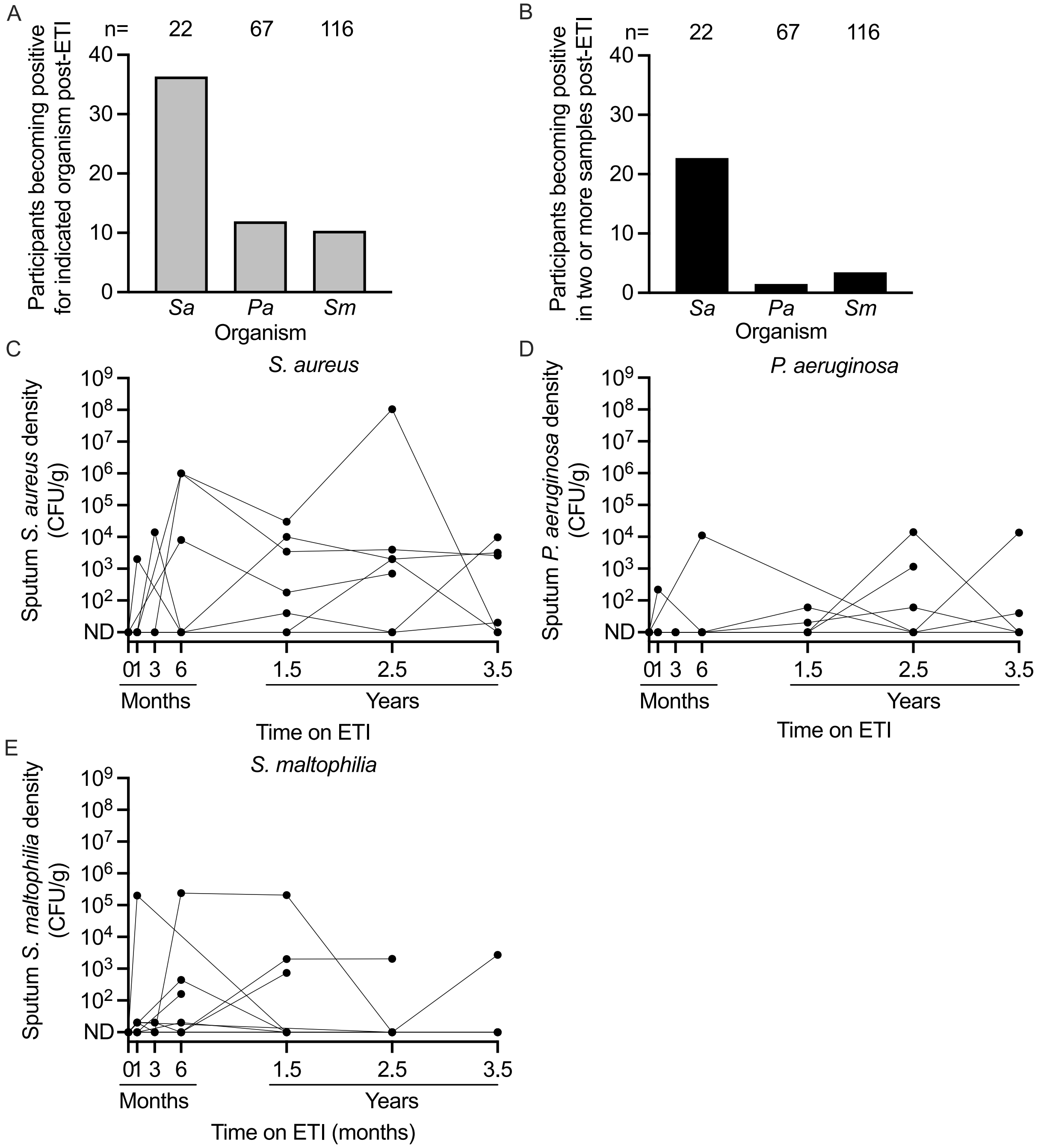

Figure S3. Participants with "new" positive cultures. Participants include in this analysis were considered negative pre-ETI if they were culture negative at the baseline visit <u>and</u> culture negative by registry report for the two-years preceding the study **A&B.** The percent of participants negative pre-ETI who had any positive cultures for indicated organisms (**A**) or at least two positive cultures **(B)** post-ETI. Participants shown in **B** are included in **A**. The number of pathogen negative participants pre-ETI is indicated above the graph. **C-E.** Culture density of indicated pathogens in these "new infections.
